# Rpf‐Toxo: A Preliminary Computationally Designed Dense Granule Antigen‐Based Multi‐Epitope Vaccine Against *Toxoplasma gondii*

**DOI:** 10.1002/vms3.71189

**Published:** 2026-08-24

**Authors:** Mohamad Hosein Safari, Seyyed Amir Hosseini, Shadan Ghiabi, Shamim Ghiabi, Davood Siamian, Hamidreza Majidiani, Hamid Irannejad, Ali Asghari

**Affiliations:** ^1^ Department of Internal Medicine Faculty of Veterinary Medicine University of Tehran Tehran Iran; ^2^ Faculty of Veterinary Medicine Science and Research Branch Islamic Azad University Tehran Iran; ^3^ Department of Medical Chemistry Faculty of Pharmacy Tehran Medical Sciences Islamic Azad University Tehran Iran; ^4^ Department of Biology Faculty of Basic Science Islamic Azad University Tonekabon Branch Iran; ^5^ Department of Basic Medical Sciences Faculty of Medicine Neyshabur University of Medical Sciences Neyshabur Iran; ^6^ Department of Medicinal Chemistry Faculty of Pharmacy Mazandaran University of Medical Sciences Sari Iran; ^7^ Drug Design and Development Research Center The Institute of Pharmaceutical Sciences Tehran University of Medical Sciences Tehran Iran; ^8^ Department of Basic Medical Sciences Khoy University of Medical Sciences Khoy Iran

**Keywords:** antigens, computer simulation, GRAs, multi‐epitope, *Toxoplasma gondii*, vaccines

## Abstract

**Background:**

*Toxoplasma gondii* is a protozoan parasite of medical and veterinary importance causing abortion. While current therapies are limited to address the chronic phase of infection, effective prophylactic vaccines are warranted.

**Objectives:**

In this study, we applied a rational vaccine design approach centred around immunodominant dense granule antigens (GRAs).

**Methods:**

In silico epitope mapping was performed on six GRAs (GRA15, GRA60, GRA76, GRA83, GRA‐α and GRA‐β). Using web servers, B‐cell, cytotoxic T‐lymphocyte and helper T‐lymphocyte epitopes were predicted and then filtered for antigenicity, solubility and allergenicity. We designed four vaccine constructs by fusing predicted epitopes with specific linkers and adjuvants (RS‐09, RpfE/50S ribosomal protein of *Mycobacterium tuberculosis*, and human interferon gamma [IFN‐γ]). Constructs were subjected to rigorous evaluation, which included physicochemical evaluation, 3D structural prediction and refinement, toll‐like receptor‐4 (TLR‐4) docking, immune simulation and codon adaptation.

**Results:**

All candidates exhibited high antigenicity (VaxiJen scores > 0.9) and solubility (> 0.6). Rpf‐Toxo emerged optimal, with non‐allergenic, antigenic (0.9148), soluble (0.658), stable (instability index: 35.41) and hydrophilic (grand average of hydropathicity: −0.641) properties. Docking revealed strong TLR‐4 binding activity (affinity: −20.6 kcal/mol; *K*_d_: 7.3e‐16 M) and immune simulation predicted robust responses, including antibody titers > 170,000, Th1‐skewed IFN‐γ (380,000 ng/mL) and memory cell activation; however, these in silico predictions need experimental validation. Codon optimization enhanced expression (CAI: 1.00; GC: 65.63%), and in silico cloning indicated compatibility with pET28a(+).

**Conclusion:**

These computational predictions require future experimental validation through in vitro and in vivo studies to confirm safety and protective efficacy.

## Graphical abstract

This graphical abstract summarizes the in silico design of the multi‐epitope vaccine candidate Rpf‐Toxo (Seq4) against *Toxoplasma gondii*. The workflow includes antigen selection, epitope mapping, vaccine construction, structural and immunological evaluation, toll‐like receptor‐4 docking, immune simulation, safety assessment, codon optimisation and *virtual* cloning, requiring further in vitro and in vivo validation.

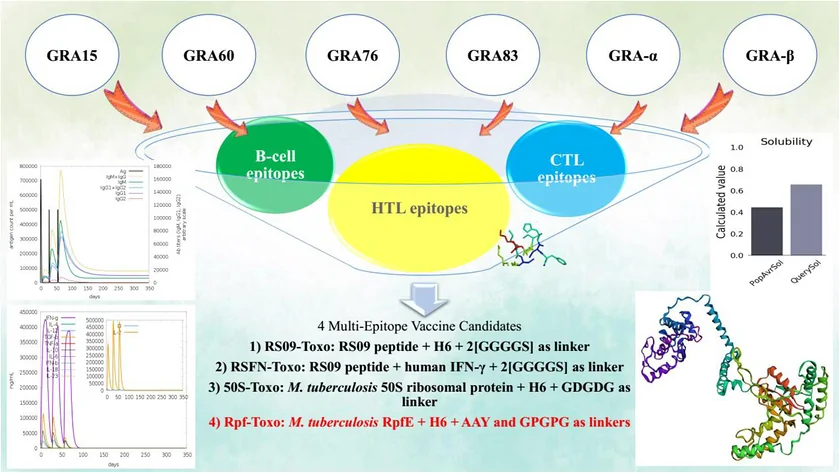

**Abbreviations**
CTL: cytotoxic T‐lymphocyte
GDT‐HA: global distance test‐high accuracy
GRA: dense granule antigen
GRAVY: grand average of hydropathicity
HLA: human leukocyte antigen
HTL: helper T‐lymphocyte
IEDB: immune epitope database
IFN‐γ: interferon gamma
I‐TASSER: Iterative Threading ASSEmbly Refinement
IVN: intravacuolar network
MEVC: multi‐epitope vaccine candidate
MHC: major histocompatibility complex
MW: molecular weight
PV: parasitophorous vacuole
PVM: parasitophorous vacuole membrane
RMSD: root mean square deviation
*T. gondii*
: *Toxoplasma gondii*
TLR‐4: toll‐like receptor‐4

## 1 Introduction

*Toxoplasma gondii* (*T. gondii*) is a widespread coccidian parasite that is found all over the world and causes toxoplasmosis in humans and in a variety of warm‐blooded animals. Felids, and particularly domestic cats, are the definitive hosts, while birds and other mammals act as intermediate hosts (Dubey 2025, Amouei et al. 2020). Toxoplasmosis can be spread via several routes, including congenital transmission, ingestion of undercooked meat containing bradyzoites and exposure to tachyzoites from blood transfusion or organ transplantation (Bollani et al. 2022, Foroutan‐Rad et al. 2016, Dard et al. 2018, Guo et al. 2015). The estimated global seroprevalence is 25.7% (95% CI: 25.6%–25.8%), with the highest burden of disease in Africa (61.4%), Oceania (38.5%) and South America (31.2%) (Molan et al. 2019).

The consequences of toxoplasmosis can be severe, especially in susceptible populations. Congenital infection can lead to chorioretinitis, hydrocephaly, microcephaly, intellectual disability and abortion (Dubey et al. 2021). In immunocompromised hosts, the parasite can cause life‐threatening inflammation of vital organs, such as the brain, heart and lungs (Huffman et al. 2022). In veterinary medicine, infection may lead to significant economic loss due to abortion and stillbirth in livestock (e.g., sheep and goats). Despite the availability of treatments aimed at the acute tachyzoite stage, including chemotherapeutic, nano‐based and herbal‐based therapies, the development of a safe and effective vaccine is necessary, since these therapies often show side effects (Dunay et al. 2018). Subunit vaccines hold promise for protecting high‐risk populations from toxoplasmosis.

Dense granule antigens (GRAs) are amongst the important antigenic proteins that *Toxoplasma* secretes upon entering the host cells. These proteins are found in association with the host cell nucleus or cytosol, the parasitophorous vacuole (PV) and its membrane (PVM), as well as the intravacuolar network (IVN), enabling the parasite to manipulate the host cell functions. Over 70 GRA proteins have been discovered in *T. gondii* so far (Rezaei et al. 2019). GRA15 is expressed in both tachyzoite and bradyzoite stages with adequate immunogenicity, inducing the host's immunity through NF‐κB pathway (Ihara et al. 2020). In recent years, it has been shown that the novel dense granule effector, GRA60, is a virulence factor of RH strain in mice acting in association with ROP18; this protein is involved in IVN and PVM during invasion (Nyonda et al. 2021). In a very recent study in 2024, GRA76 was characterized as an important virulence and growth factor for *T. gondii*, with pronounced expression in tachyzoite‐associated PVs (Zheng et al. 2024). Another novel GRA protein, GRA83, was discovered in the PV of tachyzoites and bradyzoites; it elicits innate immune responses, forcing the host to confine the parasite burden (Thind et al. 2023). Moreover, Zheng et al. (2023) discovered 15 novel *Toxoplasma* GRAs, amongst which two proteins with ToxoDB IDs TGME49_315910 and TGME49_266410, referred to as GRA‐α and GRA‐β, were shown to significantly reduce the parasite replication (both RH and Pru strains), hampering virulence and suppressing cyst formation.

Despite many years of research, there is no approved vaccine for human toxoplasmosis, though there is an approved veterinary vaccine (Toxovax) to avoid abortion in sheep (Hiszczyńska‐Sawicka et al. 2014). An effective immunogen represents a notable approach for the generation of protective innate and adaptive immunity (Zhang et al. 2013). As part of these efforts, in silico computer‐aided design of multi‐epitope vaccine candidates (MEVCs) has emerged as a powerful tool in modern vaccinology (Kelly and Rappuoli 2005, Salemi et al. 2021). Importantly, in silico approaches play an important role during early stages of the vaccine development by rapidly eliminating candidates of least favourable immunogenicity and safety. This limits the experimental burden, reducing the time, costs and ethical issues associated with inappropriate in vitro or in vivo testing, while allowing us to focus on prioritized promising candidates for further evaluation (Kelly and Rappuoli 2005, Salemi et al. 2021). Previously, several investigations used GRA proteins, particularly GRA1, GRA2, GRA5, GRA7, as a basis for mapping immunogenic epitopes and construction of MEVCs against toxoplasmosis (Rezaei et al. 2019, Ihara et al. 2020, Nyonda et al. 2021, Zheng et al. 2024, Thind et al. 2023, Zheng et al. 2023, Hiszczyńska‐Sawicka et al. 2014, Zhang et al. 2013, Kelly and Rappuoli 2005, Salemi et al. 2021, Safari et al. 2024). Our study uniquely addresses this gap by completing an extensive in silico analysis of six GRAs (GRA60, GRA76, GRA83, GRA‐α and GRA‐β) that were selected based on some criteria, such as their recent discovery as virulence factors or immunogens, having critical roles in parasite survival and/or host modulation and not being included in prior MEVCs. This was followed by the construction and assessment of a new generation of MEVCs using rigorous epitope filtering and four distinct adjuvant combinations. This method will allow an investigation of the immunogenicity of these new virulence factors in the context of their use as unprecedented vaccine components.

## 2 Materials and Methods

### 2.1 Antigen Selection and In Silico Epitope Mapping

Based on recent literature, six *T. gondii* GRA proteins were selected for their reported antigenicity, immunogenicity and/or roles in parasite virulence and characterized regarding biochemical and structural properties and their immunogenic epitopes (Tables S1 and S2). The amino acid sequences (Me49 strain) were retrieved through the ToxoDB web server, with the following IDs: TGME49_275470 (*GRA15*), TGME49_204270 (*GRA60*), TGME49_299780 (*GRA76*), TGME49_297900 (*GRA83*), TGME49_315910 (*putative GRA‐α*) and TGME49_266410 (*putative GRA‐β*). Shared continuous B‐cell epitopes were identified within each protein sequence using three web servers, including ABCpred (recurrent neural network‐based; accuracy: 65.93%), SVMTriP (fixed length: 16‐mer; SVM‐based) and ElliPro linear B‐cell epitope prediction tool (non‐fixed length; accuracy: 0.83; Thornton's method and residue clustering algorithm). The outputs from all three prediction servers for each examined GRA protein were compiled in a Microsoft Word document and cross‐compared across tools. Fragments predicted by at least two tools were retained as shared epitopes, and subsequently evaluated for antigenicity (VaxiJen v1.0), allergenicity (AllergenFP v1.0) and water solubility (PepCalc). B‐cell epitopes that demonstrated high antigenicity and solubility without allergenic potential were considered as good in silico predicted linear candidates (Table S3). For each submitted protein sequence, the top eight allele–epitope combinations (based on predefined criteria) with the lowest percentile ranks, indicating strong predicted binding affinity and broad allele representation, were selected as cytotoxic T‐lymphocyte (CTL; 9‐ or 10‐mer) epitopes associated with major histocompatibility complex (MHC) class I (Table S4) and helper T‐lymphocyte (HTL; 15‐mer) epitopes associated with MHC class II molecules (Table S5). These selected epitopes were compiled into final CTL and HTL epitope tables. CTL epitopes were subsequently evaluated for immunogenicity (immune epitope database [IEDB] tool), allergenicity (AllergenFP v1.0) and interferon gamma (IFN‐γ) induction potential (IFNepitope; hybrid approach), while HTL epitopes underwent additional screening for antigenicity (VaxiJen 2.0; accuracy: 70%–89%; target: parasite; threshold: 0.4), allergenicity, toxicity (ToxinPred) and IFN‐γ induction.

### 2.2 Design and Assemblage of Potent MEVCs

Four different MEVCs were engineered by linking different combinations of selected linear B‐cell (*n* = 6), CTL (*n* = 6) and HTL (*n* = 6) epitopes, with the latter epitopes limited to human MHCs. The constructs included one of four different adjuvants: the synthetic peptide RS‐09 (APPHALS, toll‐like receptor‐4 [TLR‐4] agonist) or, the *Mycobacterium tuberculosis* immunogens (resuscitation‐promoting factor E [RpfE] or 50S ribosomal protein), or human IFN‐γ. The functional domains were connected through the use of linkers (EAAAK, (GGGGS)_2_, GPGPG, AAY, GDGDG) in order to provide structural flexibility and independent presentation of the epitopes. A C‐terminal histidine tag (His Tag) was added to every MEVC construct, except for the construct containing both RS‐09 and IFN‐γ as adjuvants.

### 2.3 Basic Properties of the Designed MEVCs

The designed MEVCs were tested for potential allergenicity using two servers, AllerTOP v2.0 (reported accuracy of 85.3) and AllergenFP v1.0, which predicts allergens with 88% accuracy based on physicochemical and structural properties (https://ddg‐pharmfac.net/AllergenFP/) (Dimitrov et al. 2014, Zhou et al. 2026). Antigenicity was predicted with the VaxiJen v2.0 server (accuracy: 70%–89%; threshold: 0.4; target: parasite), which uses auto cross covariance transformation of protein sequence (amino acids) into uniform vectors (Doytchinova and Flower 2007, W. Wang, Liu, et al. 2026). Protein solubility post‐overexpression in *Escherichia coli* was predicted using the Protein‐Sol server (https://protein‐sol.manchester.ac.uk/); values above 0.45 were classified as soluble (Hebditch et al. 2017). Following antigenicity predictions, physicochemical characteristics (molecular weight [MW], theoretical isoelectric point or isoelectric pH, grand average of hydropathicity (GRAVY), aliphatic index and instability index) were calculated for each MEVC, using the ExPASy ProtParam tool (https://web.expasy.org/protparam/) (Gasteiger et al. 2005, W. Wang, Gao, et al. 2026).

### 2.4 Secondary and Tertiary Structures of Different MEVCs

Secondary structural properties, solvent accessibility and structural disorder were predicted on a residue‐by‐residue base using NetSurfP‐3.0 (https://services.healthtech.dtu.dk/services/NetSurfP‐3.0/) (Høie et al. 2022). Tertiary structures (three‐dimensional [3D] modelling) were predicted using the Iterative Threading ASSEmbly Refinement (I‐TASSER) server (https://zhanggroup.org/I‐TASSER/), in order to initially generate five models, based on threading templates (Yang et al. 2015, Jia et al. 2023). The model with the highest confidence score (C‐score) was selected for each construct.

### 2.5 Refining and Validating the Tertiary Model of Selected Candidate

To improve the structural quality of the chosen MEVC, the 3D model was optimized by utilizing the GalaxyRefine web server (https://galaxy.seoklab.org/cgi‐bin/submit.cgi?type = REFINE). During this procedure, a range of important quality parameters; the Clash score, the root mean square deviation (RMSD), global distance test‐high accuracy (GDT‐HA), Poor rotamers, MolProbity and Rama favored were evaluated (Heo et al. 2013). Next, the stereochemical quality of the optimized model was assessed and then compared with the crude model through Ramachandran plot processing. This assessment was completed using the PROCHECK tool in the SAVES v6.0 server (https://saves.mbi.ucla.edu/). The PROCHECK server performs a stereochemical quality assessment (both overall and local residue geometry) of a protein structure by generating multiple PostScript plots (Laskowski et al. 2006).

### 2.6 Molecular Docking Between the Best MEVC and Human TLR‐4

To conduct molecular docking analysis, the human TLR‐4 with MD‐2 complex atomic coordinates were retrieved from the RCSB Protein Data Bank using the accession code 3FXI. Protein–pprotein docking simulations were then performed using the ClusPro v.2.0 web server (https://cluspro.org/) using the default setting. This computational approach anticipated likely intermolecular amino acid contacts and affixed a binding orientation between the TLR‐4 receptor and the selected vaccine candidate (ligand). The server generated several potential complexes that were ranked based on their estimated binding energies (Kozakov et al. 2017, Koupaei et al. 2025). The binding affinity for the resulting protein–protein complexes was assessed using the online tool PRODIGY, designed to predict interaction energies in biological complexes (Xue et al. 2016, Yu‐Hao et al. 2025).

### 2.7 Prediction of Immune Response Profile

The lead vaccine candidate's immunogenic profile was simulated through C‐ImmSim (https://150.146.2.1/C‐IMMSIM/index.php), which utilizes machine learning and position‐specific scoring matrices to predict immune responses. The following parameters were set for the simulation: random seed of 12345, simulation volume of 10, and simulation steps of 1050. An immunisation schedule replicating a three‐dose schedule (1, 84 and 186 days) was employed (Rapin et al. 2011, Roohparvar Basmenj et al. 2024).

### 2.8 Conformational B‐Cell Epitope Prediction

Conformational (discontinuous) B‐cell epitopes, which are important in recognition of antigen‐antibody interactions, were determined through the ElliPro module from the IEDB analysis resource (http://tools.iedb.org/ellipro/). The analysis was performed using default parameter settings, with a minimum score of 0.5 and maximum distance of 6 (Å) (Ponomarenko et al. 2008, Xu et al. 2023).

### 2.9 Safety Prediction, Codon Adaptation and In Silico Cloning

To evaluate possible cross‐reactivity and safety, the protein sequence was assessed for homologous regions against non‐redundant protein databases using the BLASTp algorithm (https://blast.ncbi.nlm.nih.gov/Blast.cgi?PROGRAM = blastp&PAGE_TYPE = BlastSearch&LINK_LOC = blasthome) (de Araújo et al. 2025). In order to obtain a high‐yield recombinant expression, the vaccine construct was further optimized for expression in the generally‐used *E. coli* K12 strain, an organism that is well‐known for rapid growth rate, potentially high yield and ease of genetic manipulation (Fu et al. 2025). The protein sequence was first reverse‐translated to a nucleotide sequence using the Sequence Manipulation Suite. Codon optimization was then completed using the VectorBuilder tool (https://en.vectorbuilder.com/tool/codon‐optimization.html) to maximize Codon Adaptation Index (CAI) and adjust GC content, which are parameters considered highly correlated to high‐level protein expression in *E. coli*. The optimized sequence was then queried for recognition of restriction enzyme recognition sites using the NEBcutter V3.0 server (https://nc3.neb.com/NEBcutter/). Finally, in order to perform directional cloning into the pET‐28a(+) expression vector, the codon‐optimized sequence was flanked with restriction enzyme recognition sites for *Eco53kI* and *EcoRV* at the 5' and 3' ends of the construct, respectively. A Shine–Dalgarno sequence (AGGAGG) was also included upstream of the start codon to facilitate ribosomal binding and translation initiation.

## 3 Results

### 3.1 Engineering Vaccine Candidates Using Discovered Epitopes

**FIGURE 1:**
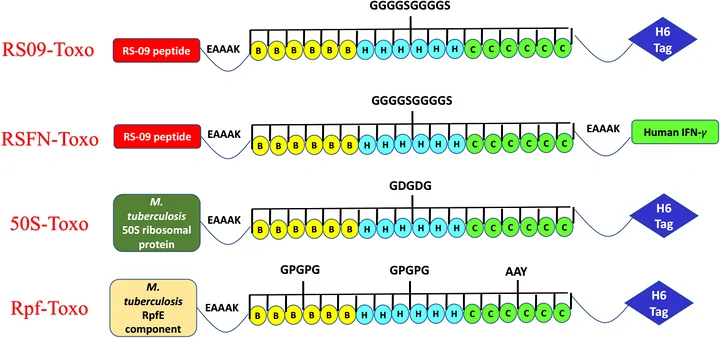
Illustration of linear B‐cell, HTL and CTL epitopes to construct four potent MEVCs using spacers, adjuvants and H6 sequences. Methodological details are described in Sections 2.1 and 2.2.

According to the in silico epitope mapping in 6 *T. gondii* GRA proteins (GRA15, GRA60, GRA76, GRA83, putative GRA‐α and putative GRA‐β), six B‐cell epitopes (GRA15_115–130_, GRA‐α_539–554_, GRA‐β_447–462_, GRA60_167–182_, GRAα_556–571_ and GRA15_134–149_), six CTL epitopes (GRA76_36–44_, GRA‐α_4–13_, GRA‐β_65–73_, GRA‐α_57–65_, GRA15_13–21_ and GRA15_71–79_) and six HTL epitopes (GRA‐α_33–47_, GRA‐α_32–46_, GRA60_52–66_, GRA60_20–34_, GRA83_65–79_ and GRA60_53–67_) were selected ultimately to construct vaccine candidates (Table 1). Four MEVCs were designed with the following epitope arrangements: *RS09‐Toxo (Seq1)*: the RS‐09 TLR‐4 agonist peptide and His‐Tag (H6) at the N‐ and C‐terminals, and epitopes linked by (GGGGS)_2_ linker; *RSFN‐Toxo (Seq2)*: a double adjuvanted polypeptide with the RS09 peptide and human IFN‐*γ* at both ends, with (GGGGS)_2_ linker; *50S‐Toxo (Seq3)*: *M. tuberculosis* 50S ribosomal protein (N‐terminal) with H6 (C‐terminal) and GDGDG as inter‐epitope linker; and *Rpf‐Toxo (Seq4)*: *M. tuberculosis* RpfE (N‐terminal), H6 (C‐terminal), with GPGPG (B‐cell and HTL) and AAY (CTL) linkers. Notably, adjuvants were linked to the sequences using the “EAAAK” linker. MEVCs (Figure 1).

**TABLE 1:** Finally selected and screened epitopes derived from the selected dense granule antigen (GRA) proteins of *Toxoplasma gondii*.

No.	B‐cell epitopes	Antigenicity^a^	HLA‐I epitopes	Immunogenicity^b^	HLA‐II epitopes	Antigenicity
**1**	GGCGADTSTDGKSESE (GRA15_115–130_)	2.6218	RTADRWLHR (GRA76_36–44_)	0.30347	DANVETENDAEDGND (GRA‐α_33–47_)	1.7223
**2**	TSQGDEDDEEEEDDDD (GRA‐α_539–554_)	2.0137	KEAEKVFEEW (GRA‐α_4–13_)	0.19685	SDANVETENDAEDGN (GRA‐α_32–46_)	1.6750
**3**	RGTVGAGGRPRGEQGK (GRA‐β_447–462_)	1.9730	EVNAVTGRF (GRA‐β_65–73_)	0.17285	NTKAFSVASEAEASG (GRA60_52–66_)	1.1178
**4**	EGTASSADSRDTGAGE (GRA60_167–182_)	1.6395	HANEPLELR (GRA‐α_57–65_)	0.15543	AGESPSAAEASVSVD (GRA‐60_20–34_)	1.0052
**5**	EADEEDGDEDEEGAQE (GRA‐α_556–571_)	1.4986	ETVPRVRTY (GRA15_13–21_)	0.15786	RAQLVTVQADPAAAT (GRA83_65–79_)	0.9485
**6**	NGEDSRFSTRTPIHVT (GRA15_134–149_)	1.4397	STSPFATRK (GRA15_71–79_)	0.14897	TKAFSVASEAEASGA (GRA60_53–67_)	0.8993

*Note*: Antigenicity and immunogenicity scores were reported to enhance the reliability of the predictions.

^a^ Indicates antigenicity using the VaxiJen v2.0 web server (threshold: 0.4).

^b^ Indicates immunogenicity using the CTL immunogenicity web tool of the IEDB web server (threshold: positive values are good immunogens).

### 3.2 Primary Biochemical Analysis of MEVCs

Based on the VaxiJen v2.0 results, all designed candidates exhibited very high antigenicity scores (> 0.9) and the highest antigenicity was observed for RS09‐Toxo with 2.1188. Also, all suggested moderately high solubility scores (above 0.6), as evidenced by the Protein‐Sol online tool. None of the candidates were allergenic, as verified by the AllerTOP and AllergenFP servers (Table 2). The length of the candidates was as follows: 451 residues (Seq1), 594 residues (Seq2), 484 residues (Seq3) and 514 residues (Seq4). The highest and lowest MWs were associated with 57036.89 (RSFN‐Toxo) and 39524.68 (RS‐09‐Toxo) Dalton, respectively. Only two candidates, that is, Seq3 (24.83) and Seq4 (35.41), were stable in the laboratory settings; also, both constructs showed good aliphatic indices with 49.77 and 46.21, respectively. All proteins were hydrophilic, as indicated by their negative GRAVY scores. Table 3 provides details of the physico‐chemical characteristics of the designed candidates.

**TABLE 2:** Prediction of antigenicity, allergenicity and solubility of novel dense granule antigen (GRA)‐based *Toxoplasma gondii* vaccine candidates.

MEVCs	Antigenicity (VaxiJen v2.0) (threshold: 0.4)	Allergenicity		Sol ability (Protein‐Sol) (threshold: 0.45)
		AllergenFP	AllerTOP	
Seq1	2.1188	No	No	0.694
Seq2	1.6747	No	No	0.777
Seq3	1.3313	No	No	0.777
Seq4	0.9148	No	No	0.658

**TABLE 3:** Physico‐chemical properties of four designed dense granule antigen (GRA)‐based vaccine candidates against *Toxoplasma gondii* infection.

MEVCs	No. of residues	MW	Theoretical pI	Positive charged residues	Negative charged residues	Half‐life	Instability index^a^	Aliphatic index^b^	GRAVY^c^
Seq1	451	39,524.68	4.14	23	65	4.4 h	60.23	24.81	−0.733
Seq2	594	57,036.89	4.65	53	85	4.4 h	50.51	39.34	−0.766
Seq3	484	48,147.08	3.92	39	126	30 h	24.83	49.77	−0.761
Seq4	514	51,155.52	4.15	33	83	30 h	35.41	46.21	−0.641

^a^ Threshold of instability index: 40; values > 40 are considered as unstable.

^b^ The higher is the aliphatic index, the wider is the thermotolerance for the protein.

^c^ Negative values indicate to hydrophilic properties in the protein.

### 3.3 Structural Evaluations of Designed MEVCs

**FIGURE 2:**
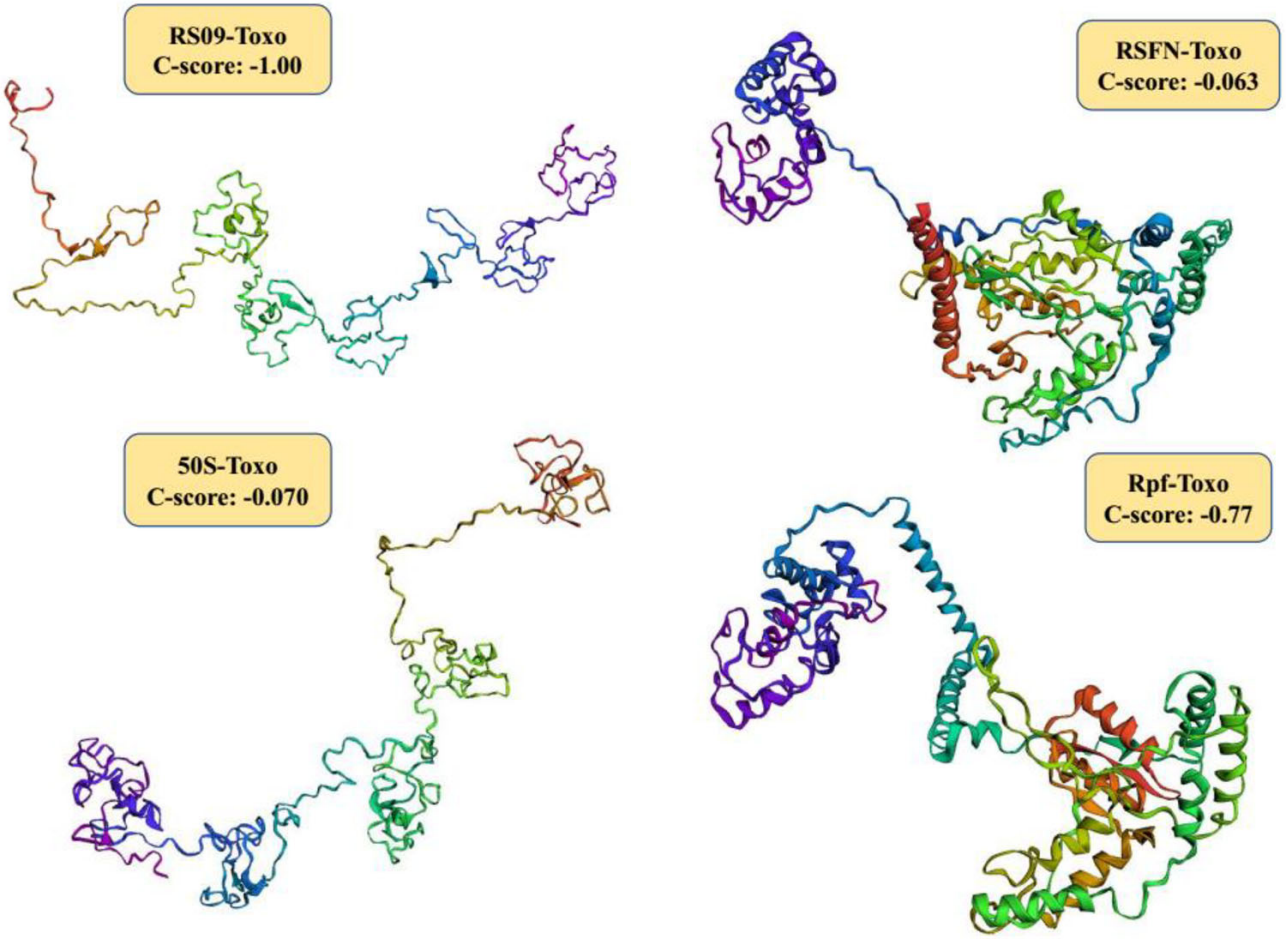
Predicted 3D models and their respective C‐scores of four designed MEVCs using the I‐TASSER web server. For methodological details, please refer to Section 2.4.

All constructs predominantly consisted of random coils, with extended strands and helices making up only a minor portion of the sequences. Only in Seq2 (RSFN‐Toxo) helices were somewhat more abundant than in other candidates, in particular C‐terminally. Disordered parts were predominant in 50S‐Toxo (positions: 1–60, 130–240, 265–305) and Rpf‐Toxo (positions: 1–95, 170–370) (Figure S1 files [S1–S4**]**). Amongst five 3D models predicted for each designed polypeptide using homology modelling server of I‐TASSER, the best models with the following C‐scores were selected: RS‐09‐Toxo model 1 (−1.00), RSFN‐Toxo model 1 (−0.63), 50S‐Toxo model 1 (−0.70) and Rpf‐Toxo model 2 (−0.77) (Figure 2).

### 3.4 Refinement and Validation of the Selected Candidate

Amongst four predicted polypeptides, one with good stability and a high aliphatic index was selected for further in silico analyses. Consequently, Seq4 (Rpf‐Toxo) was chosen as the most suitable construct and submitted to refinement analysis using the GalaxyRefine server. Accordingly, amongst five refined models provided, model #2 represented the best refined model with the subsequent parameters: 0.9721 (GDT‐HA; % of residues within structural distance thresholds, higher = better structural similarity), 0.355 (RMSD; average atomic displacement vs. ideal, lower = better refinement), 2.145 (MolProbity; composite score of steric clashes, rotamer quality and Ramachandran outliers, lower = better overall quality), 9.6 (Clash score; severe atomic steric overlaps per 1,000 atoms, lower = better), 0.3 (Poor rotamers; % of side chains in unfavorable conformations, lower = better) and 86.1 (Rama favored; % of residues in favored Ramachandran plot regions, higher = better backbone geometry). Based on the PROCHECK results, regional and global structural improvements were observed in the refined model, as compared with the crude 3D model: residues in most favored regions (70.2% vs. 61.7%%), residues in additional allowed regions (21% vs. 28.1%), residues in generously allowed regions (5.4% vs. 6.6%) and residues in disallowed regions (3.4% vs. 3.7%) (Figure S1; files [S5–S6]).

### 3.5 Vaccine Candidate—Receptor Docking

**FIGURE 3:**
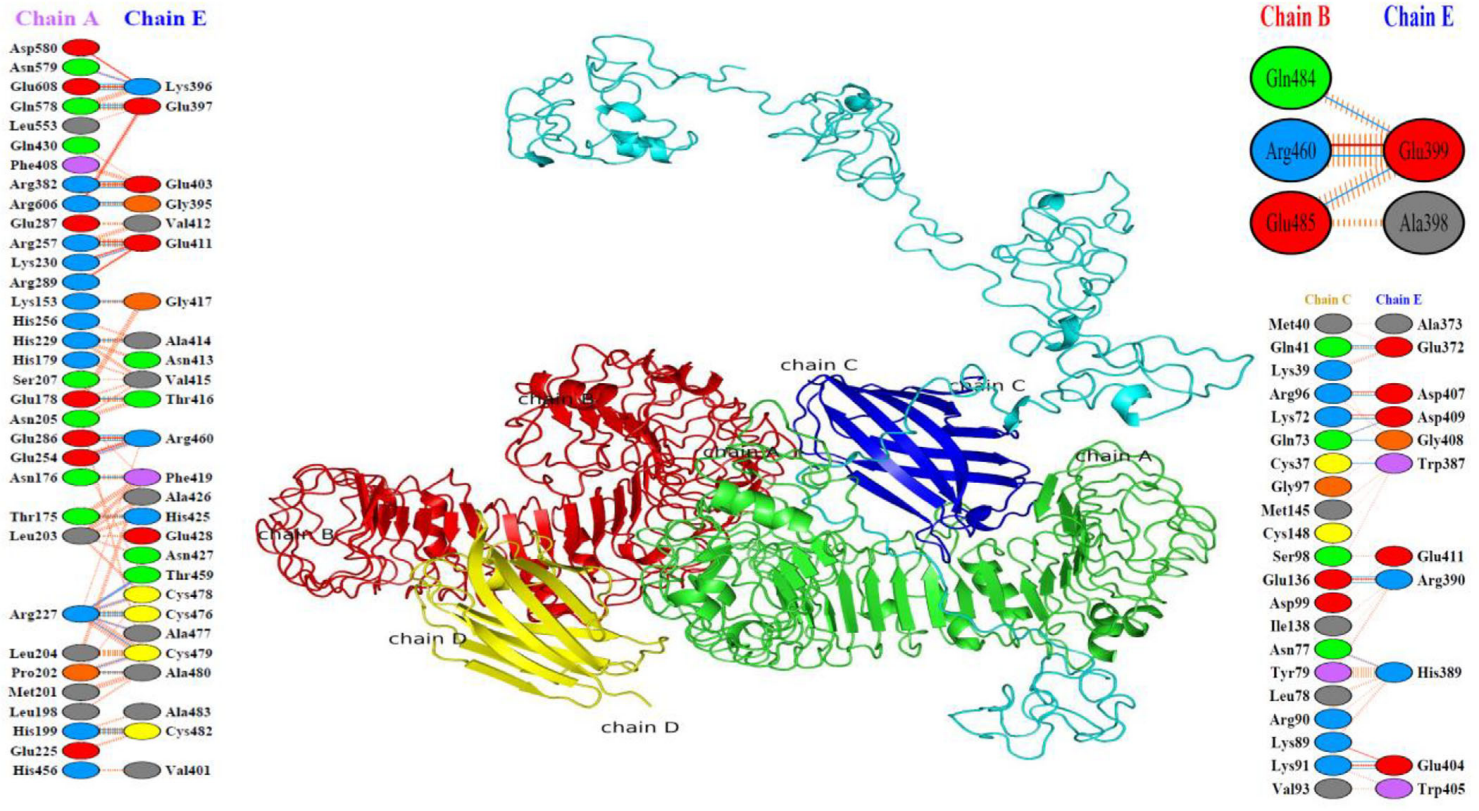
The optimal docking conformation between Rpf‐Toxo (cyan blue) when bound with chains A, B and C of the human TLR‐4 receptor is visualized in PyMol. This conformation is the lowest energy pose (score = −1424.01) of 23 cluster members. The binding energies derived from PRODIGY suggested an in silico‐predicted strong interaction energy of −20.6 kcal/mol with a *K*_d_ of 7.3 × 10^−^^1^ ^6^ M. For methodological details, please refer to Section 2.6.

The molecular docking assessment of the Rpf‐Toxo vaccine candidate with the human TLR‐4 complex (Chains A, B and C) exhibited a best‐fit complex with a stable energy quadruple score of −1424.01 and a cluster with size of 23 members (Figure 3). The selected model, displayed using PyMol (Rpf‐Toxo shown in cyan blue), was the most stable interaction predicted. The binding affinity was also evaluated using the PRODIGY server, and it was calculated to be −20.6 kcal/mol when evaluating the interaction that corresponded to a dissociation constant (*K*_d_) of 7.3 × 10^−16^ M. Although these findings may indicate strong binding affinity, such very low *K*_d_ values may reflect overestimation of prediction algorithm due to limited training dataset.

### 3.6 Simulation of Vaccine‐Derived Immune Responses

**FIGURE 4:**
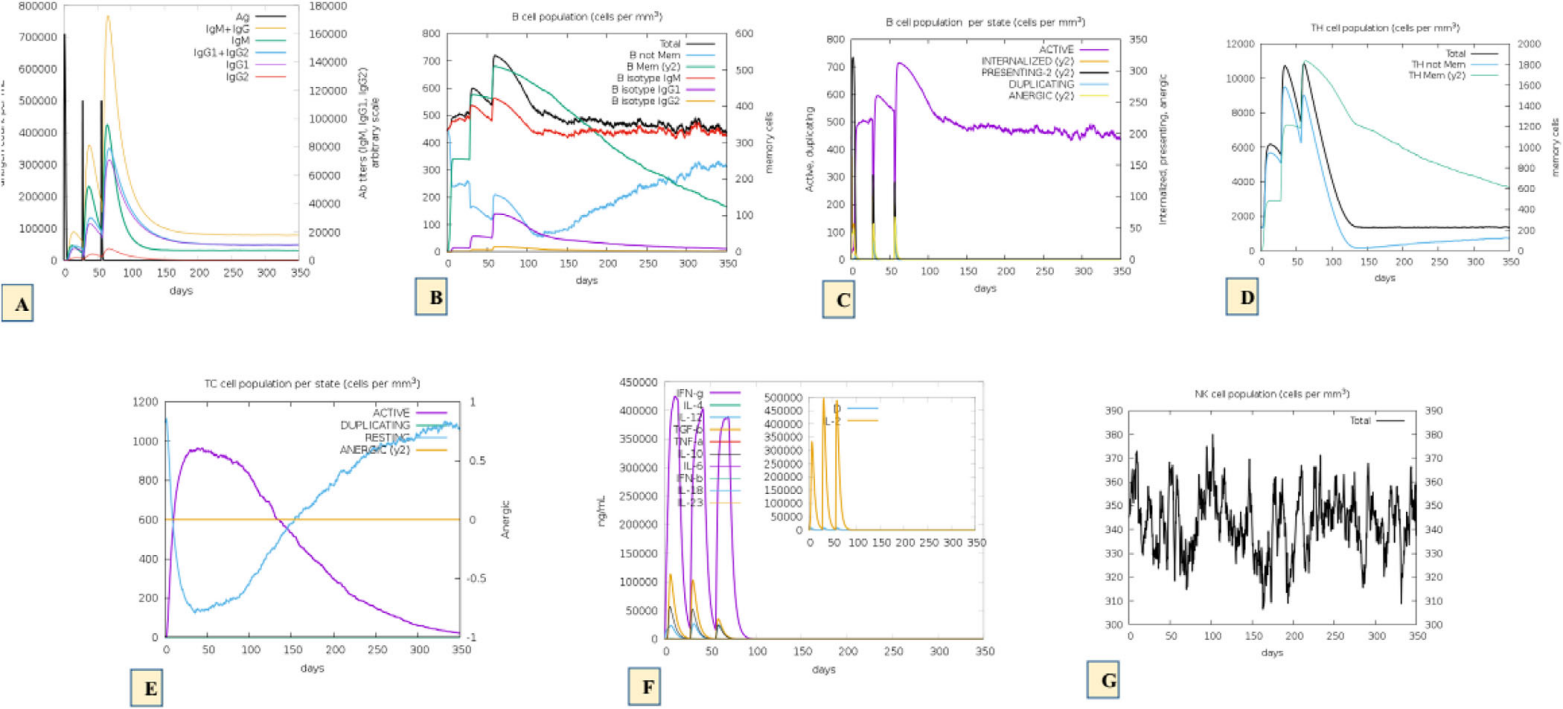
Profiles of the immune simulation upon triple vaccine candidate (Rpf‐Toxo) injection at days 1, 84 and 186, showing antibody titers (A), quantity of B‐cells per state (B and C), state of TH (D) and TC cell (E) populations, along with elicited cytokines (F) and NK cells status (G). For methodological details, please refer to Section 2.7.

For this purpose, the C‐ImmSim web server was used and the immune responses elicited upon three‐dose immunisation were evaluated. Peak IgG + IgM levels surpassed 170,000 at 65 days post‐inoculation, with maximum individual levels of IgG1 and IgM reaching approximately 70,000 and 95,000, respectively (Figure 4A). At day 60 post inoculation, the highest memory B‐cells reached 500 cells/mm^3^ and gradually declined over the subsequent 350 days. B lymphocytes isotype IgM were constant from days 100 to 350, after a minor initial increase. Additionally, B lymphocyte isotype IgG1 reached over 100 cells/mm^3^ after triple antigenic injection with a slight decrease thereafter (Figure 4B). The highest number of active B‐cells reached over 300 cells/mm^3^, then declined to about 200 cells/mm^3^ and remained constant until day 350 (Figure 4C). Upon third antigen injection, the number of active TH cells was 8000 cells/mm^3^, amongst which 1800 cells/mm^3^ were memory TH cells, until day 350 declining to below 1000 and about 600, respectively (Figure 4D). The active TC population increased by day 10 post‐inoculation (up to 900 cells/mm^3^) and remained above anergic threshold for about 140 days (Figure 4E). The peak of IFN‐γ was approximately 380,000 ng/mL, which may indicate a tendency towards Th1‐type immune response in the simulation model (Figure 4F). Of note, the peak of natural killer (NK) cells reached approximately 380 cells/mm^3^ about 100 days after last injection (Figure 4G).

### 3.7 Conformational B‐Cell Epitopes Prediction

**FIGURE 5:**
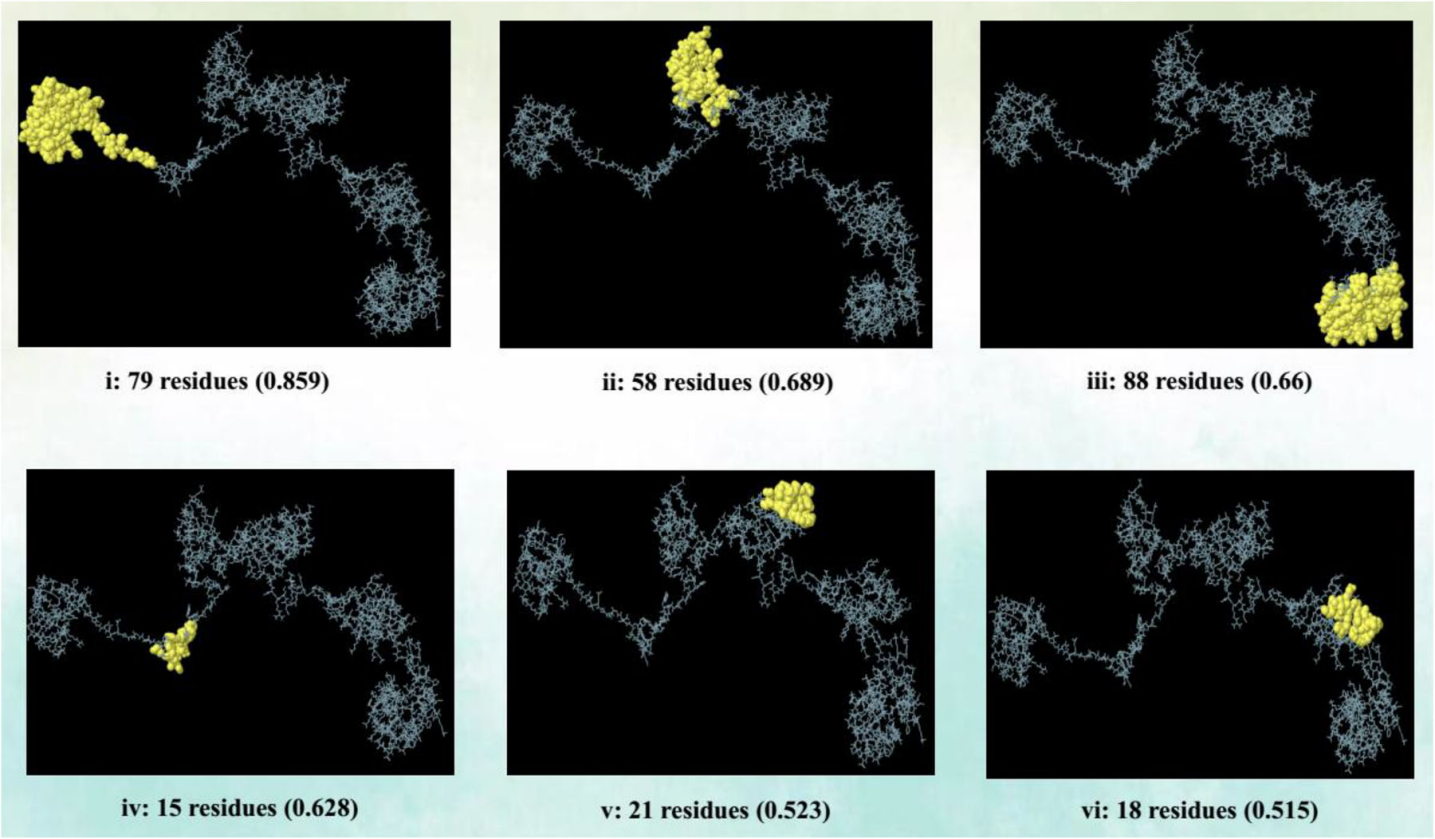
The Rpf‐Toxo (Seq4) tertiary structure is presented, with the predicted discontinuous epitopes in different colours. The epitope predictions were calculated using ElliPro (IEDB server), generating six predicted epitopes. Each epitope is numbered according to the number of amino acids, as well as an ElliPro score between 0.515 and 0.859, which indicates the probability of surface exposure and their likelihood of being antigenic conformational epitopes. Methodological details are described in Section 2.8.

Six non‐linear B‐cell epitopes were predicted in the finally selected MEVC using the ElliPro server. The number of the residues per epitope with their respective scores are given in the following: (1) 79, 0.859; (2) 58, 0.689; (3) 88, 0.66; (4) 15, 0.628; (5) 21, 0.523 and (6) 18, 0.515 (Figure 5).

### 3.8 Safety Prediction, Codon Adaptation and In Silico Cloning

In order to evaluate its safety profile, the Rpf‐Toxo amino acid sequence was screened against the human proteome using BLASTp to ensure a low probability of autoimmune cross‐reactivity, with alignment results demonstrating no significant homology. To promote optimal recombinant expression, the vaccine sequence was reverse‐translated and codon‐optimized for *E. coli* K12 strain using the VectorBuilder tool. The new sequence showed enhanced key parameters of expression, raising the CAI from a value of 0.88 to an ideal value approach of 1.00, and moved the GC content down from 70.43% to 65.63%. The new sequence was created with *Eco53kI* (GAGCTC) and *EcoRV* (GATATC) restriction sites added to the 5' and 3' terminals, respectively, to promote directional cloning into a prokaryotic expression vector. A Shine–Dalgarno sequence (AGGAGG) was placed upstream of the start codon (ATG) to increase ribosomal binding and a stop codon (TAA) was added to ensure there would be an appropriate stop to the translation.

## 4 Discussion

In the last 30 years, multiple vaccine approaches have been investigated for toxoplasmosis, including live‐attenuated, inactive, subunit and vectored strategies. The major public health goals are to prevent congenital transmission and eliminate tissue cysts (J.‐L. Wang et al. 2019). While the Toxovax live‐attenuated vaccine provides excellent protection against congenital transmission in sheep, it cannot be used in humans due to its potential subverted pathogenicity (Buxton 1993). Thus, subunit or multi‐epitope vaccines are the most beneficial candidates for humans. Rational drug design integrates structural biology, computational modelling and system pharmacology to identify molecules with the high target specificity, optimized pharmacokinetics and reduced off‐target toxicity. Advances in in silico cloning, artificial intelligence and structure‐activity relationship analysis have significantly accelerated lead optimization and translational efficiency. In parallel, modern vaccine delivery strategies leverage nanocarriers, lipid‐based systems and biomaterial scaffolds to enhance antigen stability, cellular uptake and controlled release. These delivery platforms, also, enable precise immune modulation and dose sparing, thereby improving vaccine efficacy, safety and global accessibility (Salemi et al. 2021, Parvizpour et al. 2020). Advances in reverse vaccinology and immunoinformatics have changed antigen discovery, allowing for a more rational design approach based on efficacy, stability, or safety (Donati and Rappuoli 2013). In the current study, we performed an in‐depth in silico characterization and localisation of virulence‐related GRA proteins recognized as immunogenic (GRA15, GRA60, GRA76, GRA83, GRA‐α and GRA‐β) and their peptide epitopes recognizable by humoral or cellular immunity mechanisms. In each case, four new vaccine constructs were built with optimized adjuvants and linkers and evaluated with various types of immunoinformatic tools, respectively.

This research developed four MEVCs by combining B‐cell and T‐cell epitopes using the optimized linker sequences as well as adjuvants. Each vaccine construction contained six B‐cell epitopes (GRA15_115__−__130_, GRA‐α_539__−__554_, GRA‐β_447__−__462_, GRA60_167__−__182_, GRA‐α_556__−__571_ and GRA15_134__−__149_), six human leukocyte antigen (HLA)‐I restricted CTL epitopes (GRA76_36__−__44_, GRA‐α_4__−__13_, GRA‐β_65__−__73_, GRA‐α_57__−__65_, GRA15_13__−__21_ and GRA15_71__−__79_) and six HLA‐II restricted HTL epitopes (GRA‐α_33__−__47_, GRA‐α_32__−__46_, GRA60_52__−__66_, GRA60_20__−__34_, GRA83_65__−__79_ and GRA60_53__−__67_). Population coverage for the selected epitopes was previously reported in Hosseini et al. (2025) study, where global allele‐epitope coverage estimation for predicted CTL and HTL was 88.02% and 63.86%, respectively; the highest CTL coverage was done in East Asia (93.86%), Europe (92.55%), Oceania (89.71%), the West Indies (89.34%) and North America (88.67%). This shows moderately high population coverage of the selected epitopes at the global level, particularly for CTL epitopes. The selection of a balanced mixture of B‐cell (antibody), CTL and HTL epitopes was an intentional choice to develop a broad immune response with both cellular and humoral immunity for the clearance of intracellular tachyzoites and antibody‐mediated neutralisation. The selection of linkers was important for the structural and functional integrity of each construct (Parvizpour et al. 2020). The AAY linker improves epitope presentation and acts as a proteasomal cleavage site in mammalian cells (Hasan and Mia 2022). While the GPGPG linker enhances solubility and provides flexibility between domains (Tarrahimofrad et al. 2021). The GGGGS linker is rich in glycine and serine and provides the flexibility and solubility needed for fusion proteins (Sanami et al. 2023). The KK spacer serves as a cathepsin B cleavage site required for antigen processing. Furthermore, the GDGDG linker was selected to link the B‐cell and T‐cell epitopes (Raheem et al. 2021).

To increase immunogenicity, all constructs contained adjuvants that promote Th1‐type immunity, which is important in fighting intracellular pathogens such as *T. gondii*. All constructs contained three TLR‐4 agonists. The RS‐09 synthetic peptide acts as a co‐stimulator for CTL responses (Khan et al. 2020, Coler et al. 2010, Tarang et al. 2020); however, it may ignite pro‐inflammatory cytokine production (IL‐6 and TNF‐α) at injection site, and rarely systemic responses (Dunne et al. 2011). The components of *M. tuberculosis* (RpfE and 50S ribosomal protein) allow dendritic cell maturation (Shahrear and Islam 2023). Although, RpfE possesses lower risk than immunological adjuvants (e.g., TLR agonists, IFN‐γ), it may overshadow the true responses to vaccine epitopes. The human IFN‐γ was also used for molecular adjuvant to enhance immune activation (Zanetti et al. 2023, Vivo 2000, Suárez‐Méndez et al. 2004); however, risks of immune dysregulation and expression issues are remaining (Ishii et al. 1999). The RSFN‐Toxo construct (Seq2) includes both RS‐09 and the IFN‐γ adjuvant at the N‐ and C‐termini, respectively, connected by the rigid EAAAK linker to ensure they are spatially separated from the epitope domains (Arai et al. 2001).

Final lengths of constructs were 451 (RS09‐Toxo), 594 (RSFN‐Toxo), 484 (50S‐Toxo) and 514 (Rpf‐Toxo) amino acid residues. The characterization, performed with the ProtParam server, revealed an MW between 39.52 kDa (RS09‐Toxo) and 57.03 kDa (RSFN‐Toxo), relative to length. All constructs suggested hydrophilic properties, as indicated by negative GRAVY scores. Moderate thermal stability was observed in 50S‐Toxo and Rpf‐Toxo, with aliphatic indices of 49.77 and 46.21, respectively. These two constructs also showed favourable stability, as reflected by instability index scores below the critical threshold of 40 (24.83 for 50S‐Toxo and 35.41 for Rpf‐Toxo), suggesting their suitability for laboratory use. Each vaccine construct was also predicted to be non‐allergenic using both AllerTOP v2.0 and AllergenFP v1.0 servers. Furthermore, they were also predicted to have reasonable antigenicity with VaxiJen scores, ranging between 0.9148 (Rpf‐Toxo) and 2.1188 (RS09‐Toxo), across the four constructs. Moderate solubility scores were calculated for all candidates. Although the solubility of Rpf‐Toxo (0.658) was lower than others, it is still considerably higher than the Protein‐Sol server threshold (0.45). In total, solubility, along with the instability index, balanced GC%, GRAVY score and optimal codon optimization are influencing factors in achieving high‐yield soluble expression (Bose 2022). Similar studies were conducted previously and designed MEVCs to combat *T. gondii* infection; for instance, Hammed‐Akanmu et al. (2022) designed a polytope based on B‐ and T‐cell epitopes of ROP2, MIC3 and GRA7 joined together by 50S protein L7/L12. This candidate had an MW of 51 kDa, moderate antigenicity score (0.6182) and a solubility score of 0.6182 (Hammed‐Akanmu et al. 2022). In a study by Onile et al. (2020), a polytope solely based on MIC3 was shown to possess an MW of 68.9 kDa, good solubility, aliphatic index (53.12), hydrophilicity (−0.184), with good antigenicity score of 0.8030. Forouharmehr (2021) performed an in silico epitope mapping on *T. gondii* ROP2, BiP, SOD, P30, MIC8, MIC13, GRA1, GRA2 and GRA5; he showed that the designed MEVC had an MW of 77.67 kDa, with antigenicity, GRAVY, instability and aliphatic indices of 0.92, −0.906, 29.47 and 54.12, respectively (Forouharmehr 2021). A comparison with previous GRA‐based multi‐epitope studies showed that the best selected candidate assembled by six novel GRAs in the present study (Rpf‐Toxo) suggested higher hydrophilicity, good stability and high antigenicity score, in comparison with other introduced vaccine constructs previously (Table S6) (Hammed‐Akanmu et al. 2022, Onile et al. 2020, Forouharmehr 2021, Majidiani et al. 2021, Madlala et al. 2023). Secondary structure analysis indicated that all constructs predominantly folded into random coils, which is favourablefor conformational flexibility, facilitating folding of individual epitope domains and enhanced accessibility for immune induction (J.‐L. Wang et al. 2019, Buxton 1993, Parvizpour et al. 2020, Donati and Rappuoli 2013, Hosseini et al. 2025, Hasan and Mia 2022, Tarrahimofrad et al. 2021, Sanami et al. 2023, Raheem et al. 2021, Khan et al. 2020, Coler et al. 2010, Tarang et al. 2020, Dunne et al. 2011, Shahrear and Islam 2023, Zanetti et al. 2023, Vivo 2000, Suárez‐Méndez et al. 2004, Ishii et al. 1999, Arai et al. 2001, Bose 2022, Hammed‐Akanmu et al. 2022, Onile et al. 2020, Forouharmehr 2021, Majidiani et al. 2021, Madlala et al. 2023, Buxton and Innes 1995). The I‐TASSER programme predicted tertiary structures with scores of −1.00, −0.63, −0.70 and −0.77 for each respective MEVC. The improved geometry of the Rpf‐Toxo model may indicate a more favourable structural configuration in silico, although this does not necessarily reflect its behaviour under physiological conditions. Improved backbone geometry may positively influence predicted stability and expression; however, experimental validation is required to confirm solubility, expression efficiency and immunogenic behaviour in biological systems.

Despite the strength of antigenicity and absence of allergenicity associated with all four constructs, a careful assessment of their physicochemical properties led to selection of Rpf‐Toxo for additional investigation. The RS09‐Toxo (Seq1) and RSFN‐Toxo (Seq2) constructs, while possessing the highest calculated antigenicity, had instability indices of 60.23 and 50.51, respectively, which are substantially higher than the 40 thresholds. Rapid degradation of these constructs is anticipated in vivo, ultimately eliminating them as appropriate candidates for further development. Besides Th1‐polarizing capacity, IFN‐γ adjuvant in RSFN‐Toxo (Seq2) carries several risks, including potential immunopathology, regulatory challenges, protein folding and expression issues (Rizza et al. 2011, Tovey and Lallemand 2009); hence, this candidate was disregarded. The 50S‐Toxo (Seq3) and Rpf‐Toxo (Seq4) constructs exhibited excellent stability. The use of Rpf‐Toxo over 50S‐Toxo was based on a more favourable tertiary structure prediction and refinement profile, with Rpf‐Toxo expected to be a more stable and well‐folded protein. Moreover, the adjuvant potency of *M. tuberculosis* RpfE is superior than 50S ribosomal protein, since the latter is primarily a structural carrier rather than a true immunostimulant. Although significant improvement in the Ramachandran plot (86.1%) was shown upon refinement, this remains suboptimal (> 90%) for high‐resolution crystal structures; this may result from the limitation of computational models for multi‐epitope constructs containing various flexible spacers, which naturally engage less favored regions of the Ramachandran space. The overall structural quality would be improved in future optimizations.

Simulations for the Rpf‐Toxo vaccine in the C‐ImmSim server predicted strong immunogenic response for the B‐cell activation inducing higher IgG + IgM immunoglobulin levels, increased cytokine levels of Th1‐type (IFN‐γ and IL‐2), and greater levels of memory T‐cells and NK cells on days 1, 84 and 186. Following the third immunisation, memory B‐cells peaked at 500 cells/mm^3^, then declined to approximately 200 cells/mm^3^ at day 350, rendering a stabilized memory set‐point, without loss of immunity. This is evidenced by concurrent memory Tc/Th populations, suggesting possible rapid recall responses in the simulated immune environment, suggesting a potential for sustained immune memory within the simulation model. The predicted level of IFN‐γ response (∼380,000 ng/mL at the third response) was comparable to previously published multi‐epitope vaccines using *T. gondii* ROP2, MIC3 and GRA (Hammed‐Akanmu et al. 2022). Moreover, upon single vaccine injection, our IFN‐γ level was comparable to that of Hosseini et al. (2025) and Majidiani et al. (2024) (both ∼430,000 ng/mL). The strong immunostimulatory effect observed may be attributed to the adjuvant RpfE from *M. tuberculosis*, which is known to enhance both cellular and humoral immunity. It has been shown that TLR agonists like RpfE can elevate cellular and humoral responses (Orr et al. 2014), as evidenced in multiple previous investigations (Basmenj et al. 2023, Hashemzadeh et al. 2020, Romano et al. 2012, Vakili et al. 2018, Zhao et al. 2015). Note that these results are only in silico and are not indicative of real immune protection in complex in vivo microenvironment.

Conformational B‐cell epitopes are important targets for antigen‐antibody interactions (Ansari and Raghava 2010, W. Wang et al. 2025); accordingly, six epitopes were predicted in the current study for the Rpf‐Toxo candidate. The molecular docking study was carried out via ClusPro 2.0 using human TLR‐4 receptor. This receptor is implicated in provoking innate immune responses against intracellular pathogens, including *T. gondii*, which is associated in the literature with Th1‐biased dominance of pro‐inflammatory cytokines (IFN‐γ and IL‐12) for parasite elimination. Moreover, TLR‐4 is a critical receptor expressed on dendritic cells and macrophages, which are central to antigen presentation and adaptive immune activation (Debierre‐Grockiego et al. 2007, Egan et al. 2009, Munoz et al. 2011). Strong binding of Rpf‐Toxo to chain A of human TLR‐4 was predicted through 241 non‐bonded interactions, 30 hydrogen bonds and 9 salt bridges. Nevertheless, the estimated *K*_d_ (7.3 × 10^−16^), which suggests potential binding affinity predicted in silico, appears to be an overestimation of the PRODIGY server, which introduces uncertainty. Since static structures are only considered in docking simulation without the impact of solvation, conformational flexibility and dynamic protein–protein interactions in natural condition (Alonso et al. 2006, Naqvi et al. 2018), interpretation of these findings should be accompanied with caution. Overall, docking results only suggest possible interactions in silico and do not imply functional immune activation in vivo. Such limitations of computational tools underscore the need for future experimental validation using TLR‐4‐specific reporter assays and in vivo challenge studies. An important finding was that BLASTp analysis showed no significant homology to any human proteins present in the sequence of the vaccine to ensure human safety. The vaccine sequence was codon‐optimized for *E. coli* K12 strain using VectorBuilder to improve the transcriptional and translational efficiency A 65.63% GC content was achieved upon codon optimization; however, protein expression efficiency may be affected by increased stability of mRNA secondary structure, biased codon usage and/or transcriptional pausing (Francis and Page 2010). Nevertheless, a CAI value of 1.00 and > 70 GC content still may partially mitigate these concerns. The resulting sequence also included *Eco53kI* and *EcoRV* restriction sites at the 5' and 3' terminus, right before a Shine–Dalgarno ribosomal binding site and a start and stop codon to facilitate easy cloning and expression in pET‐28a^+^ and similar vectors for recombinant production and eventual immunological testing in future studies. In silico codon optimization and CAI improvement only suggest theoretical expression efficiency and must be confirmed experimentally in expression systems such as *E. coli* BL21(DE3).

Our study met some limitations, including (1) prediction of immunogenicity, safety profiles, as well as protection efficacy of the designed vaccine constructs (RS09‐Toxo, RSFN‐Toxo and 50S‐Toxo, Rpf‐Toxo) remain theoretical, until validated using in vitro and in vivo experiments; (2) the correct identification of B‐cell, CTL and HTL epitopes, as well as the predicted physicochemical properties (i.e., antigenicity, allergenicity, solubility and stability), 3D structures and binding affinity for TLR‐4, suffered from algorithmic bias and dataset quality dependency, which may render false positive results and/or inconsistent performance across different pathogens. All bioinformatics tools have their own biases and limitations that could influence the results; (3) while the simulated immune responses are useful, these responses are too simplified to fully recapitulate the variations seen in a mammalian host in vivo, including the specific amplitude, kinetics, or qualitative aspects of immune activation; (4) while partial binding affinity to TLR‐4 was predicted, this does not confer functional biological activity and subsequently effective downstream immune signaling activation; (5) while different adjuvants were suggested here, the synergistic effects and circumstantial risks associated with these adjuvants remains theoretical; (6) conservation analysis of the selected epitopes across diverse *T. gondii* strains was not performed, which is essential to evaluate the potential for broad cross‐protection against genetically distinct isolates. This will be addressed in future studies; (7) MD simulation evaluating the stability and dynamic behaviour of protein–protein complex was not feasible in the current study due to time and equipment limitations; (8) generalisation of the predicted immune responsiveness across different HLA alleles worldwide could not be achieved, due to lack of population coverage analysis; and (9)  for enhanced translational efficiency and transcript stability, mRNA secondary structure analysis is essential, though was not feasible to conduct in this study. Finally, the findings in the present study should be validated through wet lab experiments to reach a more precise conclusion on the potency of GRA‐based epitopes in immune induction against *T. gondii*.

## 5 .Conclusion

This study was entirely based on computational predictions and in silico analyses, which, despite their growing applicability in vaccine design processes, inherently lack the complexity of in vivo systems. Immunogenicity, stability and protein expression of the designed MEVCs were predicted using simulation‐based web tools, which, as a result, do not reflect the biological behaviour of living systems. In the present study, only two out of four designed MEVCs had stable profiles, according to the ProtParam analysis, amongst which Rpf‐Toxo was shown as the best candidate to be assessed in future experimental studies. Additionally, immune simulation and molecular docking were performed under ideal circumstances that do not conform to the population variabilities, such as MHC polymorphisms and individual immune status. Although the Rpf‐Toxo construct provided attractive immunoinformatics qualities, adding several linked epitopes and adjuvants may have complications related to multiepitope vaccines. Epitope competition may happen if adjacent domains sterically hinder immune recognition, or immune masking may limit the exposure of important epitopes. While flexible linkers were included (e.g., GPGPG and AAY) to reduce interference, it is essential to confirm that all epitopes maintain the original immunogenicity without cross‐reactive suppression. Thus, it is necessary to further validate these findings, such as protein expression and immunogenicity, in animal studies in order to assess the vaccine candidate's safety, efficacy, feasibility and biological relevance to the at‐risk population.

### 5.1 Current and Future Developments

The Rpf‐Toxo construct represents an immunoinformatics‐derived MEVC that warrants further experimental evaluation. It is important to emphasise that all findings are based on in silico predictions. No experimental immunological validation, including cytokine assays, expression studies, or animal challenge models, was performed. Therefore, the proposed construct should be considered a theoretical vaccine candidate requiring further biological confirmation. Next steps should focus on MD simulation analysis of the proposed vaccine‐TLR4 complex stability, advanced delivery platforms (liposomes, nanoparticles), molecular docking simulations for dynamic vaccine‐ligand interactions and experimental validation in murine challenge models. Ultimately, continued collaboration between computational and translational scientists will be a necessity to generate a safe, effective vaccine capable of preventing congenital toxoplasmosis and eliminating tissue cysts.

## Author Contributions

Conceptualization: Mohamad Hosein Safari, Seyyed Amir Hosseini, Hamidreza Majidiani, and Ali Asghari. Methodology: Ali Asghari, Hamidreza Majidiani, Seyyed Amir Hosseini. Formal analysis and investigation: Mohamad Hosein Safari, Seyyed Amir Hosseini, Shadan Ghiabi, Shamim Ghiabi, Davood Siamian, Hamid Irannejad. Writing – original draft preparation: Mohamad Hosein Safari, Shadan Ghiabi, Shamim Ghiabi, Davood Siamian, Hamidreza Majidiani. Writing – review and editing: Ali Asghari and Hamidreza Majidiani. Supervision: Hamidreza Majidian and Ali Asghari. All authors read and approved the final version of the manuscript.

## Funding

The authors have nothing to report.

## Conflicts of Interest

The authors declare no conflicts of interest.

## Supporting information




**Supplementary Table S1**: Prediction of the physicochemical characteristics of some important GRAs of *T. gondii* as novel vaccine antigens.

**Supplementary Table S2**: Prediction of the antigenicity, allergenicity, and solubility of several important GRAs of *T. gondii* as novel vaccine antigens

**Supplementary Table S3**: Shared linear B‐cell epitopes of the selected GRAs (GRA15, 60, 76, 83, α, and β) screened in terms of antigenicity, allergenicity, and water solubility.

**Supplementary Table S4**: Predicted CTL epitopes of the selected GRAs (GRA15, 60, 76, 83, α, and β) screened in terms of immunogenicity, allergenicity, and IFN‐γ induction.

**Supplementary Table S5**: Predicted HTL epitopes of the selected GRAs (GRA15, 60, 76, 83, α, and β) screened in terms of antigenicity, allergenicity, toxicity, and IFN‐γ induction.

**Supplementary Table S6**: Comprehensive comparison of published GRA‐based multi‐epitope vaccine constructs against *T. gondii*.

**Supplementary Fig. S1**: Secondary structure prediction for all designed candidates along with the Ramachandran plot analysis for Seq4 (selected candidate).

## Data Availability

The data that support the findings of this study are available from the corresponding author upon reasonable request.
