# Supplementary material for: Rpf‐Toxo: A Preliminary Computationally Designed Dense Granule Antigen‐Based Multi‐Epitope Vaccine Against Toxoplasma gondii
Source: Vet Med Sci. 2026 Aug 24;12(5):e71189. doi: 10.1002/vms3.71189 (PMC13504016; doi:10.1002/vms3.71189)
Supplement: Supplementary file 1 [file VMS3-12-e71189-s001.docx]

**Supplementary Table S1.** Prediction of the physicochemical characteristics of some important GRAs of *T. gondii* as novel vaccine antigens.

| **New vaccine candidate antigens** | **Number of amino acids** | **Molecular weight (MW)** | **Theoretical isoelectric point (pI)** | **Negatively charged residues (Asp+Glu)** | **Positively charged residues (Arg+Lys)** | **The estimated half-life in mammalian cells (hours)** | **The instability index** | **Aliphatic index** | **Grand average of hydropathicity (GRAVY)** |
| --- | --- | --- | --- | --- | --- | --- | --- | --- | --- |
| **GRA15** | 550 | 57890.03 | 9.16 | 44 | 48 | 30 | 66.06 | 59.45 | -0.573 |
| **GRA60** | 544 | 58107.14 | 9.82 | 55 | 68 | 30 | 64.21 | 71.67 | -0.480 |
| **GRA76** | 545 | 59371.23 | 9.54 | 44 | 59 | 30 | 36.23 | 92.50 | -0.049 |
| **GRA83** | 763 | 82719.84 | 9.56 | 72 | 93 | 30 | 48.46 | 87.88 | -0.198 |
| **Novel GRA-α** | 575 | 62716.64 | 4.07 | 140 | 47 | 30 | 60.79 | 63.01 | -0.797 |
| **Novel GRA-β** | 586 | 63916.32 | 5.53 | 89 | 79 | 30 | 50.12 | 89.76 | -0.324 |
