# Supplementary material for: Rpf‐Toxo: A Preliminary Computationally Designed Dense Granule Antigen‐Based Multi‐Epitope Vaccine Against Toxoplasma gondii
Source: Vet Med Sci. 2026 Aug 24;12(5):e71189. doi: 10.1002/vms3.71189 (PMC13504016; doi:10.1002/vms3.71189)
Supplement: Supplementary file 2 [file VMS3-12-e71189-s002.docx]

**Immunodominant fragments from dense granule antigens (GRAs) of *Toxoplasma gondii*: Pieces of a puzzle in vaccine design**

**Supplementary Figure S1.** Secondary structure of RS09-Toxo vaccine construct (**Seq1**):

**Supplementary Figure S2.** Secondary structure of RSFN-Toxo vaccine construct (**Seq2**):

**Supplementary Figure S3.** Secondary structure of 50S-Toxo vaccine construct (**Seq3**):

**Supplementary Figure S4.** Secondary structure of Rpf-Toxo vaccine construct (**Seq4**):

**Supplementary Figure S5.** Ramachandran plot analysis of the crude vaccine model (**Rpf-Toxo or** **Seq4**):

**Supplementary Figure S6.** Ramachandran plot analysis of the refined vaccine model (**Rpf-Toxo or** **Seq4**):
