# Supplementary material for: Rpf‐Toxo: A Preliminary Computationally Designed Dense Granule Antigen‐Based Multi‐Epitope Vaccine Against Toxoplasma gondii
Source: Vet Med Sci. 2026 Aug 24;12(5):e71189. doi: 10.1002/vms3.71189 (PMC13504016; doi:10.1002/vms3.71189)
Supplement: Supplementary file 3 [file VMS3-12-e71189-s003.docx]

**Supplementary Table S2.** Prediction of the antigenicity, allergenicity, and solubility of several important GRAs of *T. gondii* as novel vaccine antigens

| **No.** | **New vaccine candidate antigens** | **ToxoDB Accession** | **Antigenicity score (VaxiJen v2.0)** | **Allergenicity** | | | **Solubility**  **(Protein-Sol)** |
| --- | --- | --- | --- | --- | --- | --- | --- |
|  |  |  |  | **AlgPred 2 Hybrid** | **AllergenFP** | **AllerTOP** |  |
| **1.** | GRA15 | TGME49_275470 | 0.6961 | No (score: 0.05) | No | No | 0.423 |
| **2.** | GRA60 | TGME49_204270 | 0.7243 | No (score: 0.02) | No | No | 0.421 |
| **3.** | GRA76 | TGME49_299780 | 0.5812 | No (score: 0.04) | No | No | 0.230 |
| **4.** | GRA83 | TGME49_297900 | 0.4885 | No (score: 0.05) | No | No | 0.338 |
| **5.** | Novel GRA-α | TGME49-315910 | 0.7412 | No (score: 0.18) | No | No | 0.817 |
| **6.** | Novel GRA-β | TGME49-266410 | 0.5595 | No (score: 0.02) | No | No | 0.554 |
