# Supplementary material for: Rpf‐Toxo: A Preliminary Computationally Designed Dense Granule Antigen‐Based Multi‐Epitope Vaccine Against Toxoplasma gondii
Source: Vet Med Sci. 2026 Aug 24;12(5):e71189. doi: 10.1002/vms3.71189 (PMC13504016; doi:10.1002/vms3.71189)
Supplement: Supplementary file 4 [file VMS3-12-e71189-s004.docx]

**Supplementary Table S4.** Cytotoxic T-lymphocyte (CTL) specific epitope prediction for some novel GRAs of *T. gondii* against IEDB HLA reference set and subsequent screening regarding immunogenicity and IFN-γ induction

| **Protein** | **Alleles from HLA reference set** | **Start-End** | **CTL epitope** | **Percentile rank** | **Immunogenicity** | **Allergenicity** | **IFN-γ induction** |
| --- | --- | --- | --- | --- | --- | --- | --- |
| ***T. gondii* GRA15** | HLA-B*35:01 | 43-51 | IPSPGTNTF | 0.01 | 0.0219 | Yes | Negative |
|  | HLA-A*26:01 | 13-21 | ETVPRVRTY | 0.01 | 0.15786 | Yes | **Positive** |
|  | HLA-A*24:02 | 22-30 | VYQLNPHVF | 0.01 | -0.01408 | Yes | Negative |
|  | HLA-A*11:01 | 71-79 | STSPFATRK | 0.01 | 0.14897 | No | **Positive** |
|  | HLA-B*53:01 | 43-51 | IPSPGTNTF | 0.03 | 0.0219 | Yes | Negative |
|  | HLA-B*57:01 | 45-54 | SSKSRSIIRW | 0.01 | -0.07933 | Yes | Negative |
|  | HLA-A*23:01 | 22-30 | VYQLNPHVF | 0.02 | -0.01408 | Yes | Negative |
|  | HLA-B*07:02 | 60-68 | GPRDTQSAV | 0.02 | -0.14888 | Yes | Negative |
| ***T. gondii* GRA60** | HLA-B*07:02 | 70-79 | SPRNRVPSSL | 0.01 | -0.14583 | Yes | Negative |
|  | HLA-B*07:02 | 34-42 | RPRSVLPLL | 0.01 | -0.13575 | Yes | Negative |
|  | HLA-B*07:02 | 39-47 | APSSLRVNL | 0.01 | -0.15119 | Yes | Negative |
|  | HLA-B*57:01 | 47-56 | RETVDDIMRW | 0.01 | 0.10466 | Yes | Negative |
|  | HLA-B*58:01 | 47-56 | RETVDDIMRW | 0.01 | 0.10466 | Yes | Negative |
|  | HLA-A*03:01 | 14-22 | SMSDPQLFK | 0.04 | -0.09218 | Yes | Negative |
|  | HLA-B*57:01 | 56-64 | VTLALWLLW | 0.02 | 0.21764 | No | Negative |
|  | HLA-A*68:01 | 48-57 | ETVDDIMRWR | 0.01 | 0.19472 | No | Negative |
| ***T. gondii* GRA76** | HLA-A*68:01 | 28-36 | ETWDTLVKR | 0.01 | 0.03042 | Yes | Negative |
|  | HLA-A*68:01 | 25-33 | EVVRVQAFR | 0.01 | 0.09806 | Yes | **Positive** |
|  | HLA-A*31:01 | 36-44 | RTADRWLHR | 0.01 | 0.30347 | No | **Positive** |
|  | HLA-A*68:01 | 30-38 | DSISPGAER | 0.01 | -0.01065 | Yes | Negative |
|  | HLA-A*11:01 | 9-17 | GSQAPVFRK | 0.01 | 0.15887 | No | Negative |
|  | HLA-A*11:01 | 41-49 | SVYDMFDTK | 0.01 | 0.00172 | Yes | Negative |
|  | HLA-B*15:01 | 58-67 | VMREGTNLVY | 0.02 | 0.19702 | Yes | Negative |
|  | HLA-A*03:01 | 42-50 | RLREHVEKK | 0.04 | 0.14641 | Yes | **Positive** |
| ***T. gondii* GRA83** | HLA-B*07:02 | 40-48 | TPARLRQAL | 0.01 | 0.0278 | No | Negative |
|  | HLA-A*01:01 | 2-10 | AIDTVTLLY | 0.01 | 0.10716 | Yes | Negative |
|  | HLA-A*01:01 | 28-36 | AADSQSWTY | 0.01 | -0.21818 | No | Negative |
|  | HLA-B*40:01 | 12-20 | GEEEEVFVL | 0.01 | 0.39253 | Yes | Negative |
|  | HLA-B*57:01 | 20-28 | VGNPVSKHW | 0.04 | -0.29189 | Yes | Negative |
|  | HLA-B*15:01 | 18-26 | IQARIAAAY | 0.01 | 0.28709 | Yes | Negative |
|  | HLA-A*31:01 | 30-38 | ATKQPQVQR | 0.01 | -0.33924 | Yes | Negative |
|  | HLA-A*24:02 | 35-44 | TYTPVVAHAF | 0.01 | 0.16883 | No | Negative |
| ***T. gondii* novel GRA-α** | HLA-B*57:01 | 38-46 | VTGRLSLRW | 0.01 | -0.08257 | Yes | Negative |
|  | HLA-B*57:01 | 42-50 | LSLRWRRQW | 0.01 | 0.2889 | Yes | Negative |
|  | HLA-B*58:01 | 38-46 | VTGRLSLRW | 0.01 | -0.08257 | Yes | Negative |
|  | HLA-A*02:06 | 22-30 | KVMQEVEEV | 0.01 | 0.1058 | No | Negative |
|  | HLA-A*68:01 | 57-65 | HANEPLELR | 0.02 | 0.15543 | Yes | **Positive** |
|  | HLA-B*44:02 | 4-13 | KEAEKVFEEW | 0.01 | 0.19685 | Yes | Positive |
|  | HLA-B*44:03 | 4-13 | KEAEKVFEEW | 0.02 | 0.19685 | Yes | **Positive** |
|  | HLA-B*07:02 | 10-18 | APHPPPSPA | 0.03 | -0.168 | Yes | **Positive** |
| ***T. gondii* novel GRA-β** | HLA-B*40:01 | 20-28 | VEFPRPTTL | 0.01 | 0.12224 | Yes | **Positive** |
|  | HLA-B*57:01 | 65-73 | ESLRKFFEW | 0.03 | 0.10598 | No | Negative |
|  | HLA-A*68:01 | 11-20 | TTLKDITTKR | 0.01 | -0.1261 | No | Negative |
|  | HLA-B*57:01 | 39-47 | MSLGKMLMF | 0.04 | -0.45676 | Yes | Negative |
|  | HLA-B*07:02 | 49-58 | SPRSRVGTTV | 0.03 | 0.02827 | No | Negative |
|  | HLA-B*40:01 | 8-17 | QEAENVVNQL | 0.03 | 0.11307 | Yes | Negative |
|  | HLA-B*40:01 | 46-54 | IESRALEQL | 0.03 | 0.04286 | Yes | Negative |
|  | HLA-A*26:01 | 65-73 | EVNAVTGRF | 0.01 | 0.17285 | Yes | **Positive** |

*** indicates potentially qualified epitopes.**
