# Supplementary material for: Rpf‐Toxo: A Preliminary Computationally Designed Dense Granule Antigen‐Based Multi‐Epitope Vaccine Against Toxoplasma gondii
Source: Vet Med Sci. 2026 Aug 24;12(5):e71189. doi: 10.1002/vms3.71189 (PMC13504016; doi:10.1002/vms3.71189)
Supplement: Supplementary file 5 [file VMS3-12-e71189-s005.docx]

**Supplementary Table S3.** Shared continuous B-cell epitopes predicted for the *T. gondii* GRAs using three different web servers (ABCpred, SVMTriP, and ElliPro); they were screened regarding antigenicity, allergenicity and water solubility.

| **Protein** | **Epitope Sequence** | **Antigenicity (VaxiJen server)** | **Allergenicity (AllergenFP server)** | **Solubility (PepCalc server)** |
| --- | --- | --- | --- | --- |
| **GRA15** | ERTISDPGPQRAGAAQ | 0.0945 | No | Good |
|  | FGPQQTPPWMDETAAA | -0.4312 | No | Poor |
|  | AAIPLAPSQPGSRTQP | 0.7245 | Yes | Poor |
|  | HIETEARLTYSNHLKS | 1.1119 | Yes | Good |
|  | IIGAGSSA | 0.9951 | Yes | Poor |
|  | SAVPGPPRTENPRQPQ | 0.7438 | Yes | Good |
|  | APATATSNHMHASATP | 0.7567 | No | Poor |
|  | ALDFIFPHTP | -0.2145 | Yes | Poor |
|  | LPVVENATFFGPQQTP | 0.1946 | No | Poor |
|  | GGCGADTSTDGKSESE | 2.6218 | No | Good |
|  | PLSQQETLPVVENATF | -0.1711 | No | Poor |
|  | RFSTRTPIHVT | 1.4226 | No | Good |
|  | RRVSRDLSDSVRETRR | 0.5383 | No | Good |
|  | NGEDSRFSTRTPIHVT | 1.4397 | No | Good |
| **GRA60** | VASSYLPRGMDERQRP | 0.5975 | Yes | Good |
|  | SMKSQW | -0.6853 | No | Good |
|  | RVPSSLPETSMKSQW | 0.3853 | No | Good |
|  | EGTASSADSRDTGAGE | 1.6395 | No | Good |
|  | KRFFGNPKQMRFRAQE | 0.9707 | Yes | Good |
|  | ASLVSSPLTKTSDQA | 0.7768 | No | Good |
|  | RETVDDIMRWREYLRP | 0.2246 | Yes | Good |
|  | RRVSVASEGRGEGRTE | 1.7741 | Yes | Good |
|  | LSRVQTPSFGNYAATV | 0.2329 | Yes | Poor |
|  | LPLLGHEGTASSADSR | 0.9847 | Yes | Good |
|  | DRETVDDIMRWREYLR | 0.1675 | Yes | Good |
|  | TSTSLTSP | 1.1019 | Yes | Poor |
|  | QSLLASPRNRVPSSLP | 0.9487 | No | Good |
| **GRA76** | DNGIVTT | 0.9458 | Yes | Good |
|  | LGRIPERRRERFGVVN | 0.3921 | No | Good |
|  | TWDTLVKRTADRWL | -0.5936 | No | Good |
|  | GILLRGGGLHGAAATS | 1.0832 | No | Poor |
|  | HVEKKEGSTVIK | 0.2725 | Yes | Good |
|  | AERGQARLREHVE | 0.9537 | Yes | Good |
|  | EALSQMGIDRHHGSV | -0.3472 | No | Good |
|  | LACVSPGHQPVFFLNP | 0.6940 | No | Poor |
|  | DDEGPTAYHFAP | 0.4307 | Yes | Good |
|  | LGLLVRGCTHDIRRGV | 0.9391 | No | Good |
|  | LKSLKHRAIYAVLSVI | 0.3536 | Yes | Poor |
|  | HVEVVRVQAFRSLQ | 0.0659 | No | Good |
|  | NVELTVDAVGILACVS | 1.0623 | No | Poor |
|  | AALVSLGLLVRGCTHD | 1.1729 | No | Poor |
|  | TFVLEATL | 0.7128 | Yes | Poor |
| **GRA83** | HSKASAHGPSDSPATK | 1.1004 | Yes | Good |
|  | SGARLRGIGADAGLDN | 0.4499 | No | Good |
|  | RGPGRHA | 1.2203 | Yes | Good |
|  | QHRCGDDVRFKTGDKP | -0.2443 | No | Good |
|  | HKSDPPKADVP | 0.5332 | Yes | Good |
|  | EGEPILSVVNRTTG | -0.1362 | No | Good |
|  | PRTEGRGERGP | 1.6466 | Yes | Good |
|  | PAAATKKGERVAHRAQ | 0.6289 | No | Good |
|  | LPAQSDKTQLRK | 0.4182 | No | Good |
|  | SETGFVDVVKVH | -0.3392 | No | Good |
|  | SETQGAVEAVTAKLTA | 0.5849 | Yes | Good |
|  | PQNTSAFKHPIAKL | 0.3446 | No | Poor |
|  | IVATMRNKGLHSRVRM | 1.1327 | No | Good |
|  | DVKTFFAALGYLVNSA | 0.8743 | Yes | Poor |
| **GRA-α** | TQAEETGTANAAQ | 0.9676 | Yes | Good |
|  | ASIAFGEPDQLMDV | 0.1279 | No | Good |
|  | SVLETLTT | 0.9063 | Yes | Poor |
|  | EADEEDGDEDEEGAQE | 1.4986 | No | Good |
|  | TSQGDEDDEEEEDDDD | 2.0137 | No | Good |
|  | MSSQTTRGVASAKRSK | 0.8575 | No | Good |
|  | PRAGPVETESDANVET | 1.1400 | No | Good |
|  | GDGEKAAVESF | 0.7760 | No | Good |
|  | ASVHQKTSLMDAMKM | 0.1918 | No | Good |
|  | CADVTATPAPHPPPSP | 0.3956 | Yes | Poor |
|  | LRSLQSLFCFVAM | -0.4105 | No | Poor |
|  | ETENDAEDGNDATDQT | 1.3934 | Yes | Good |
|  | APENVQTPEQLPRAGP | -0.1518 | No | Good |
|  | FPEVAKRKSMIY | -0.7955 | Yes | Good |
| **GRA-β** | GGRRRGAVEEDNVEFP | 0.3597 | No | Good |
|  | RGTVGAGGRPRGEQGK | 1.9730 | No | Good |
|  | QVSDDALEDSDAKAEI | 0.9086 | No | Good |
|  | MCDVIDRIENDTNDAR | 0.2415 | No | Good |
|  | NSAIDKATTLKDITTK | 0.0456 | No | Good |
|  | LSVIDELNSAIDKATT | -0.5750 | No | Good |
|  | ARSSAGGPLRFADLFP | 0.6616 | No | Good |
|  | FFEWLMSERP | 0.6564 | Yes | Good |
|  | EAAGREAEDRRAKIAA | 1.3779 | No | Good |
|  | VHLASVEVASLVGDVD | 0.6763 | No | Poor |
|  | VMLSVIDELNSAIDKA | -0.3038 | No | Good |
|  | RIANFVGDSGVTLAVV | 0.1423 | Yes | Poor |
|  | DARSNLVILEEKLKDA | 1.1246 | No | Good |
|  | TARVMLKQL | 0.3096 | No | Good |
