# Supplementary material for: Rpf‐Toxo: A Preliminary Computationally Designed Dense Granule Antigen‐Based Multi‐Epitope Vaccine Against Toxoplasma gondii
Source: Vet Med Sci. 2026 Aug 24;12(5):e71189. doi: 10.1002/vms3.71189 (PMC13504016; doi:10.1002/vms3.71189)
Supplement: Supplementary file 6 [file VMS3-12-e71189-s006.docx]

**Supplementary Table S6.** Comprehensive comparison of published GRA-based multi-epitope vaccine constructs against *Toxoplasma gondii*

| **Study** | **Year** | **Used GRAs** | **Other Ags** | **Adjuvant** | **Length (aa)** | **MW (kDa)** | **Antigenicity score** | **Instability index** | **Aliphatic index** | **GRAVY** | **Validation level** |
| --- | --- | --- | --- | --- | --- | --- | --- | --- | --- | --- | --- |
| Current study (Rpf-Toxo) | 2026 | GRA15, GRA60, GRA76, GRA83, GRA-α, GRA-β | None | *M. tuberculosis* RpfE | 514 | 51.16 | 0.9148 | 35.41 | 46.21 | -0.641 | *In silico* |
| Madlala et al. | 2023 | GRA7 | AMA1, ROP16 | Profilin | ~660 | 73.35 | 0.6645 | 28.70 | 64.10 | -0.363 | *In silico* + MD simulation |
| Hammed-Akanmu et al. | 2022 | GRA7 | ROP2, MIC3 | 50S L7/L12 | 469 | 50.35 | 0.6182 | 37.51 | 77.08 | -0.439 | *In silico* + MD simulation |
| Forouharmehr | 2021 | GRA1, GRA2, GRA5 | BiP, MIC8, MIC13, P30, PI1, SOD, ROP2 | HBHA (*M. tuberculosis*) | 730 | 77.67 | 0.92 | 29.47 | 54.12 | NR | *In silico* only |
| Majidiani et al. | 2021 | GRA12 | SAG1, ROP16, GRA12, MIC4 | None (Vector-based vaccine) | 282 | 28.06 | 0.7547 | 0.9592 | 44.57 | -0.375 | *In silico* + *In vivo* |

**NR:** Not reported | **MD:** Molecular Dynamics | **HBHA:** Heparin-binding hemagglutinin | **RpfE:** Resuscitation-promoting factor E
