# Supplementary material for: Rpf‐Toxo: A Preliminary Computationally Designed Dense Granule Antigen‐Based Multi‐Epitope Vaccine Against Toxoplasma gondii
Source: Vet Med Sci. 2026 Aug 24;12(5):e71189. doi: 10.1002/vms3.71189 (PMC13504016; doi:10.1002/vms3.71189)
Supplement: Supplementary file 7 [file VMS3-12-e71189-s007.docx]

**Supplementary Table S5.** Helper T-lymphocyte specific epitope prediction for some novel GRAs of *T. gondii* against HLA reference set with subsequent screening regarding antigenicity, allergenicity, toxicity, and IFN-γ induction

| **Protein** | **Alleles from HLA reference set** | **Start-End** | **HTL epitope** | **Percentile rank** | **Antigenicity** | **Allergenicity** | **Toxicity** | **IFN-γ induction** |
| --- | --- | --- | --- | --- | --- | --- | --- | --- |
| ***T. gondii* GRA15** | HLA-DRB1*04:01 | 28-42 | ENSYYSVPTERTISD | 0.13 | 0.1800 | No | No | **Positive** |
|  | HLA-DQA1*01:02/DQB1*06:02 | 53-67 | MDETAAAAIPLAPSQ | 0.14 | 0.2632 | No | No | Negative |
|  | HLA-DRB1*07:01 | 39-53 | RARRVASSKSRSIIR | 0.15 | 0.6638 | No | No | **Positive** |
|  | HLA-DRB1*07:01 | 35-49 | IDPVVGTSGHSVAVG | 0.15 | 0.8146 | No | No | **Positive** |
|  | HLA-DRB1*07:01 | 34-48 | SIDPVVGTSGHSVAV | 0.19 | 0.4328 | No | No | **Positive** |
|  | HLA-DRB1*12:01 | 19-33 | FDVVYQLNPHVFRSR | 0.22 | 0.2134 | Yes | No | **Positive** |
|  | HLA-DRB1*09:01 | 39-53 | RARRVASSKSRSIIR | 0.24 | 0.6638 | No | No | Positive |
|  | HLA-DRB1*07:01 | 40-54 | ARRVASSKSRSIIRW | 0.24 | 0.0817 | No | No | **Positive** |
| ***T. gondii* GRA60** | HLA-DRB1*13:02 | 22-36 | QSQFRLSPRSSRRPR | 0.02 | 0.5263 | No | No | **Positive** |
|  | HLA-DQA1*05:01/DQB1*03:01 | 21-35 | GESPSAAEASVSVDG | 0.02 | 1.4405 | No | No | Negative |
|  | HLA-DRB1*09:01 | 53-67 | TKAFSVASEAEASGA | 0.03 | 0.8993 | No | No | **Positive** |
|  | HLA-DRB1*13:02 | 21-35 | RQSQFRLSPRSSRRP | 0.04 | 0.3809 | No | No | Negative |
|  | HLA-DRB1*09:01 | 52-66 | NTKAFSVASEAEASG | 0.04 | 1.1178 | No | No | **Positive** |
|  | HLA-DQA1*05:01/DQB1*03:01 | 20-34 | AGESPSAAEASVSVD | 0.05 | 1.0052 | No | No | **Positive** |
|  | HLA-DQA1*05:01/DQB1*03:01 | 17-31 | SRGAAAGSVETAGDS | 0.07 | 1.5007 | No | No | Negative |
|  | HLA-DRB1*07:01 | 51-65 | QRPILSTVASLVSSP | 0.08 | 0.4643 | No | No | Negative |
| ***T. gondii* GRA76** | HLA-DRB1*01:01 | 60-74 | AQGYVLQTAEQVVLT | 0.02 | 0.6455 | No | No | Negative |
|  | HLA-DRB1*01:01 | 37-51 | PDTFVLEATLPIRRN | 0.02 | 0.8612 | No | No | **Positive** |
|  | HLA-DRB1*09:01 | 32-46 | MNPYNMAAAAGVSLA | 0.05 | 0.1333 | No | No | Negative |
|  | HLA-DRB1*15:01 | 8-22 | AGLIGIYYGIRGAKR | 0.05 | -0.0105 | Yes | No | Negative |
|  | HLA-DRB1*01:01 | 59-73 | NAQGYVLQTAEQVVL | 0.05 | 0.6931 | Yes | No | Negative |
|  | HLA-DPA1*02:01/DPB1*05:01 | 58-72 | LNAQGYVLQTAEQVV | 0.05 | 0.8397 | No | No | Negative |
|  | HLA-DRB5*01:01 | 37-51 | PDTFVLEATLPIRRN | 0.06 | 0.8612 | No | No | Positive |
|  | HLA-DRB1*09:01 | 11-25 | IGIYYGIRGAKRQIT | 0.06 | 0.1984 | No | No | Negative |
| ***T. gondii* GRA83** | HLA-DQA1*03:01/DQB1*03:02 | 19-33 | QARIAAAYVAADSQS | 0.03 | 0.6136 | No | No | **Positive** |
|  | HLA-DQA1*03:01/DQB1*03:02 | 18-32 | IQARIAAAYVAADSQ | 0.04 | 0.6685 | No | No | **Positive** |
|  | HLA-DRB5*01:01 | 33-47 | ALGSFIPTPARLRQA | 0.04 | 0.8119 | No | No | Negative |
|  | HLA-DRB5*01:01 | 34-48 | LGSFIPTPARLRQAL | 0.06 | 0.9562 | No | No | Negative |
|  | HLA-DRB4*01:01 | 65-79 | RAQLVTVQADPAAAT | 0.08 | 0.9485 | No | No | **Positive** |
|  | HLA-DQA1*03:01/DQB1*03:02 | 43-57 | GGNVEGVVNEREQTQ | 0.09 | 1.5885 | No | No | Negative |
|  | HLA-DRB1*15:01 | 5-19 | NAGLGVFPALGIGNR | 0.09 | 0.8688 | Yes | No | **Positive** |
|  | HLA-DQA1*01:02/DQB1*06:02 | 3-17 | FLITRGVVIARGAVE | 0.1 | -0.3344 | No | No | Negative |
| ***T. gondii* novel GRA-α** | HLA-DQA1*03:01/DQB1*03:02 | 33-47 | DANVETENDAEDGND | 0.01 | 1.7223 | No | No | **Positive** |
|  | HLA-DQA1*03:01/DQB1*03:02 | 32-46 | SDANVETENDAEDGN | 0.06 | 1.6750 | Yes | No | **Positive** |
|  | HLA-DQA1*05:01/DQB1*02:01 | 33-47 | DANVETESDADVETE | 0.09 | 1.7223 | No | No | Negative |
|  | HLA-DQA1*05:01/DQB1*02:01 | 33-47 | DANVETESDADVETE | 0.09 | 1.7223 | No | No | Negative |
|  | HLA-DQA1*05:01/DQB1*02:01 | 33-47 | DANVETESDADVETE | 0.13 | 1.7223 | No | No | Negative |
|  | HLA-DQA1*05:01/DQB1*02:01 | 33-47 | DANVETESDADVETE | 0.13 | 1.7223 | No | No | Negative |
|  | HLA-DQA1*05:01/DQB1*02:01 | 33-47 | DANVETESDADVETE | 0.13 | 1.7223 | No | No | Negative |
|  | HLA-DQA1*05:01/DQB1*02:01 | 33-47 | DANVETESDADVETE | 0.13 | 1.7223 | No | No | Negative |
| ***T. gondii* novel GRA-β** | HLA-DQA1*01:02/DQB1*06:02 | 23-37 | DLQNVHATAAEATKR | 0.01 | 0.0110 | No | No | **Positive** |
|  | HLA-DQA1*01:02/DQB1*06:02 | 22-36 | IDLQNVHATAAEATK | 0.03 | 0.0195 | Yes | No | **Positive** |
|  | HLA-DRB1*01:01 | 12-26 | NEEIKRLEAERAALQ | 0.05 | 0.1237 | No | No | Negative |
|  | HLA-DQA1*01:02/DQB1*06:02 | 24-38 | LQNVHATAAEATKRE | 0.08 | 0.2844 | No | No | Negative |
|  | HLA-DQA1*01:02/DQB1*06:02 | 59-73 | VVEDKAAVLEAAGRE | 0.08 | 0.3180 | No | No | **Positive** |
|  | HLA-DRB1*01:01 | 11-25 | TNEEIKRLEAERAAL | 0.11 | 0.1031 | No | No | Negative |
|  | HLA-DRB5*01:01 | 66-80 | VVSYLLVQNRKRIAN | 0.12 | 0.3017 | No | No | Negative |
|  | HLA-DRB3*01:01 | 56-70 | SEGVVEDKAAVLEAA | 0.14 | 0.7986 | No | No | Negative |

*** indicates potentially qualified epitopes.**
